# Successful Thrombolysis despite Having an Incidental Unruptured Cerebral Aneurysm

**DOI:** 10.1155/2014/323049

**Published:** 2014-11-24

**Authors:** Diana Briosa e Gala, André Almeida, Nadine Monteiro, Ana Paiva Nunes, Patrícia Ferreira, Nuno Mendonça, Alexandre Amaral-Silva, João Alcântara

**Affiliations:** ^1^Internal Medicine Department, Centro Hospitalar Leiria-Pombal, EPE, Hospital de Santo André, 2410-197 Leiria, Portugal; ^2^Internal Medicine Department, Centro Hospitalar de Lisboa Central, EPE, Hospital de Santa Marta, 1169-024 Lisboa, Portugal; ^3^Internal Medicine Department, Centro Hospitalar de Lisboa Ocidental, EPE, Hospital Egas Moniz, 1349-019 Lisboa, Portugal; ^4^Cerebrovascular Unit, Centro Hospitalar de Lisboa Central, EPE, Hospital de São José, 1150-199 Lisboa, Portugal

## Abstract

*Purpose*. To report a case of successful thrombolysis performed in a patient with an incidental unruptured intracranial aneurysm and review the literature. *Case Report*. Patient admitted for ischemic stroke due to left posterior cerebral artery occlusion, with an incidental right middle cerebral artery aneurysm, who underwent treatment with tissue plasminogen activator (rtPA) resulting in clinical improvement without complications. *Conclusion*. The presence of unruptured intracranial aneurysms is considered as a contraindication to thrombolysis, due to a potentially higher hemorrhagic risk of aneurysm rupture. Patients, otherwise, eligible for thrombolysis are usually excluded from receiving this emergent treatment, despite its potential benefits. A reevaluation of the strict exclusion criteria for thrombolysis in acute stroke patients should be considered.

## 1. Introduction

Therapeutic guidelines for intravenous thrombolysis for treating hyperacute ischemic stroke are very strict [1]. Evidence of unruptured cerebral aneurysms contraindicates thrombolysis due to the perceived increase in the risk of rupture and consequent intracranial hemorrhage as a result of rtPA administration [1, 2].

## 2. Case Presentation

A patient was admitted to the emergency room eighty minutes after the onset of sudden left conjugated ocular deviation with right homonymous hemianopia, right central facial palsy and hemiparesis, as well as right hypoesthesia without neglect. The initial National Institute of Health Stroke Scale (NIHSS) score was 16 points.

An initial noncontrast-enhanced brain computer tomography (CT) study showed a spontaneous hyperdensity on the P2 segment of the left posterior cerebral artery. There was no evidence of other major vessel occlusion or signs of intracranial hemorrhage; therefore, recombinant tissue plasminogen activator (rtPA) was administered intravenously, at a dosage of 0.9 mg/Kg, 92 minutes after the onset of symptoms.

**Figure 1:**
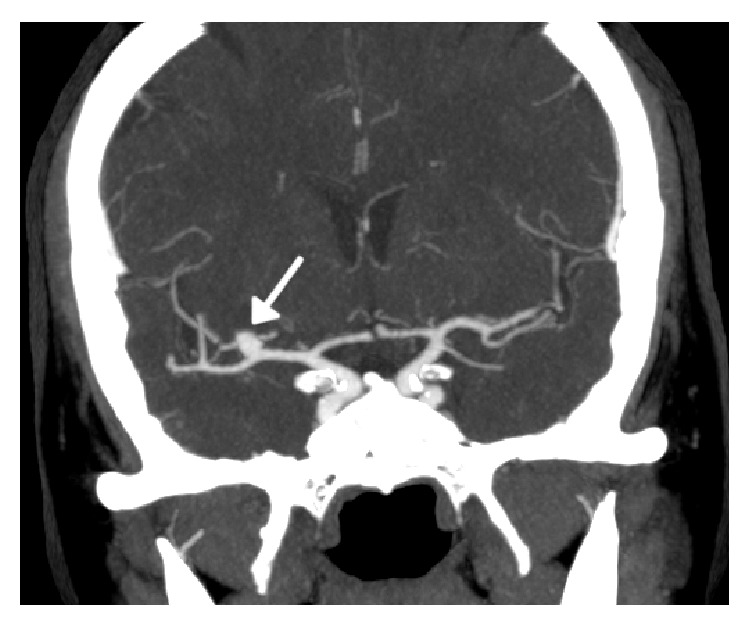
7 mm saccular aneurysm at the right sylvian trifurcation.

A CT angiogram performed during rtPA perfusion confirmed a left posterior cerebral artery occlusion at the P1-P2 transition and a 7 mm saccular aneurysm at the right sylvian trifurcation was detected (Figure 1). The patient underwent immediate brain diffusion magnetic resonance imaging (MRI) and sentinel bleeding due to the fact that aneurysm rupture was excluded and a left thalamic acute ischemic lesion was confirmed. After weighing the available clinical data and the sum of the neuroimaging studies it was decided to maintain intravenous (IV) thrombolysis.

Following IV thrombolysis, significant clinical improvement occurred, with a NIHSS of 6 points at the 24-hour time point.

A brain CT performed 24 h after thrombolysis did not show hemorrhagic complications.

An angiography was performed eight days after thrombolysis. This confirmed the presence of a saccular aneurysm with a diameter of 5 mm at the right middle cerebral artery.

Complete aneurysm exclusion was accomplished using a low-profile visualized intraluminal support (LVIS) stent plus embolization with coils. There were no complications related to this procedure.

A brain CT performed before discharge identified a recent left thalamus-capsular ischemic infarct and signs of aneurysm embolization.

The patient was discharged scoring 4 in the NIHSS and 2 in the modified Rankin scale.

## 3. Discussion

Intravenous tissue-type plasminogen activator is the only Food and Drug Administration approved pharmacological therapy for patients with an acute ischemic stroke [2].

A clinically important intracranial hemorrhage (ICH) can develop as an adverse effect of thrombolytic therapy and is the most feared complication of thrombolysis [3].

Some risk factors for bleeding after the administration of rtPA have been either documented or proposed. One of the risks is the presence of unruptured cerebral aneurysms, thus resulting in this risk being listed as a contraindication.

Theoretically, there is an increased postthrombolysis ICH risk from aneurysm rupture, as it is known that rtPA alters vascular permeability as well as the integrity of vascular basal lamina [4]. However, how significant this risk is has not been established [5].

The exclusion criterion for performing thrombolysis in the presence of unruptured cerebral aneurysms was designated before initial trials, thereby establishing no relation between the size and location of the aneurysms and lacking clear definition as an exclusion criterion in many protocols [6].

The incidences of unruptured cerebral aneurysms and subarachnoid hemorrhage suggest that most intracranial aneurysms do not rupture [7]. However, data on aneurysm prevalence and risk of aneurysm rupture vary according to study design, study population, aneurysm size and location, history of previous aneurysm rupture, and the neuroimaging techniques used for its detection [7, 8]. In patients without any previous SAH, the annual risk of rupture of small aneurysms, measuring <10 mm, is low (0.5% per year) [9]. Symptomatic aneurysms, aneurysms located in the posterior circulation, including basilar artery aneurysms, aneurysms harbored by patients with a previous SAH from a separate intracranial aneurysm, and aneurysms larger than 10 mm, have a markedly increased risk of rupture [8].

In consideration of prethrombolysis neuroimaging techniques in protocols where a standard CT without contrast agents is the only exam required, cerebral aneurysms, particularly unruptured intracranial aneurysms, cannot be adequately excluded, resulting in a subsequent underreporting of cerebral aneurysms real prevalence [9].

Taking into account the implementation of recent neuroimaging techniques, there has been an increase in the incidental discovery of aneurysms in patients, otherwise, eligible for thrombolysis [5]. The exclusion of an early hemorrhage from aneurysm rupture is a key point in the decision of whether or not to continue thrombolysis in these patients.

To date, several cases have been reported of safe IV administration of rtPA in patients with cerebral aneurysms which were either previously known or incidentally discovered when undergoing intracranial angiogram (computed tomography, magnetic resonance, or catheter angiography) during the first hours after stroke [1, 10].

Nevertheless, a few cases have been described where cerebral aneurysm rupture with ICH developed after IV thrombolysis, in one case administered for treatment of myocardial infarction [1] and in two others for ischemic stroke. Regarding the latter, the stroke was related to a left middle cerebral artery thromboembolism and thrombolysis resulted in an anterior communicating artery aneurysm rupture [11]. In another study, CT angiography performed in the setting of clinical deterioration which followed thrombolysis demonstrated that a likely left internal carotid artery dissection with middle cerebral artery thromboembolism had been the event behind the patient's initial presentation [12].

Four retrospective cohort studies on this issue have been conducted in centers with dedicated stroke units, compounding a total of 714 patients who received IV thrombolysis for acute stroke, 48 of whom had intracranial unruptured aneurysms. All of these either did not show a significant difference in the rate of bleeding as a result of rtPA administration among patients with aneurysms compared to the rate among those without [5, 13, 14] or failed to establish an association between the use of thrombolysis and aneurysm rupture [15].

## 4. Conclusion

This case report suggests that intravenous rtPA administered in patients with acute ischemic stroke in the presence of unruptured cerebral aneurysms may, in selected cases, have a good benefit-to-risk ratio. Notwithstanding the limited time frame for acute stroke treatment, the decision to maintain thrombolysis in patients with unreported intracranial aneurysms must, following a step-by-step approach, consider all available data. This approach allows the expansion and improvement of the use of thrombolysis in these patients, while safely considering a delayed treatment of the aneurysms.

Despite some reports in the literature of successful off-label thrombolysis cases in this group of patients, there may still be significant underreporting of cases similar to ours which could contribute to a modification of the current strict guidelines for acute ischemic stroke [1].

We suggest recording intracranial aneurysms in the prospective registries of thrombolysis in acute stroke patients.
